# Connexin 43 Phosphorylation: Implications in Multiple Diseases

**DOI:** 10.3390/molecules28134914

**Published:** 2023-06-22

**Authors:** Meng Zhang, Zhen-Zhen Wang, Nai-Hong Chen

**Affiliations:** State Key Laboratory of Bioactive Substances and Functions of Natural Medicines, Institute of Materia Medica & Neuroscience Center, Chinese Academy of Medical Science and Peking Union Medical College, Beijing 100050, China

**Keywords:** connexin 43, phosphorylation, intercellular communication, the cardiovascular system, the nervous system

## Abstract

Connexin 43 (Cx43) is most widely distributed in mammals, especially in the cardiovascular and nervous systems. Its phosphorylation state has been found to be regulated by the action of more than ten kinases and phosphatases, including mitogen-activated protein kinase/extracellular signaling and regulating kinase signaling. In addition, the phosphorylation status of different phosphorylation sites affects its own synthesis and assembly and the function of the gap junctions (GJs) to varying degrees. The phosphorylation of Cx43 can affect the permeability, electrical conductivity, and gating properties of GJs, thereby having various effects on intercellular communication and affecting physiological or pathological processes in vitro and in vivo. Therefore, clarifying the relationship between Cx43 phosphorylation and specific disease processes will help us better understand the disease. Based on the above clinical and preclinical findings, we present in this review the functional significance of Cx43 phosphorylation in multiple diseases and discuss the potential of Cx43 as a drug target in Cx43-related disease pathophysiology, with an emphasis on the importance of connexin 43 as an emerging therapeutic target in cardiac and neuroprotection.

## 1. Introduction

Connexins are proteins that are broadly distributed throughout the body and are crucial for heart and brain health. At the plasma membrane, six connexin subunits come together to form a linker or hemichannel. Gap junction channels (GJCs) are created as a result of the two hemichannels interacting with one another in a head-to-head configuration [1]. Gap junctions supply the intracellular communications that are linked to prolonged physiological processes, cellular development, and stability [2,3,4]. These communications are also associated with the rapid exchange of cations, transmitters, and second messengers, which are fundamental atoms for electrophysiological excitability and its transmission. The regulation of intracellular Ca^2+^ mobilization and K^+^ buffering, in particular, helps the astroglial gap junction contribute to the cytoplasm-to-cytoplasm communication of biochemical or ionic mobilization between a cell and adjacent cells, resulting in the regulation of ionic and several other types of homeostasis [5]. Through the formation of functioning syncytial networks in the central nervous system (CNS), astrocytes serve crucial homeostatic roles. Compared to other connexins, such as Cx26, Cx30, and Cx32, connexin 43 (Cx43), which is expressed by astrocytes, is the most prevalent connexin in the brain and is one of the key components of gap junctions [6]. Similarly, Cx43 is also the most prominent connexin in the ventricles, which forms gap junctions and hemichannels and is also present in the mitochondria of the heart [7]. Thus, it plays an important role in the cardiovascular system and the nervous system.

Approximately 80% of astrocyte coupling in the hippocampus is mediated by Cx43, which has at least 21 phosphorylation sites on its C-terminus [8]. Protein kinase C (PKC) has been demonstrated to phosphorylate Cx43 at Ser368 and Ser372 in vitro, and tissue polypeptide antigen (TPA) has been shown to promote Ser262 and Ser368 phosphorylation [9]. Ser368 phosphorylation has also been demonstrated to be responsible for the TPA-induced decrease in intercellular communication. Purified recombinant Cx43 was phosphorylated by PKC, which eliminated sucrose and Lucifer yellow permeability and caused a conformational shift in Cx43. In cardiomyocytes, PKC was discovered to connect with Cx43. In lens epithelial cells, PKC binds with Cx43 [10]. Fibroblast growth factor-2 increased the colocalization of PKC with Cx43 via increasing Cx43 phosphorylation and decreasing cardiomyocyte gap junctional conductance [11]. It has been demonstrated that casein kinase 1 (CK1) interacts with and phosphorylates Cx43, especially the isoform [12]. There are other kinases that undoubtedly phosphorylate Cx43, and it indicates that different kinases can phosphorylate the same position. For instance, mitogen-activated protein kinase (MAPK) has been observed to phosphorylate Ser255 [13]. Therefore, a large body of evidence suggests that Cx43 is a highly phosphorylated and tightly controlled protein.

**Figure 1:**
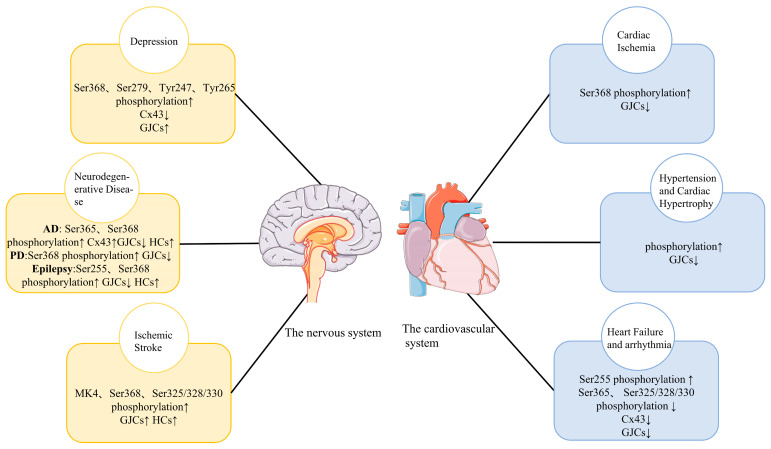
Changes in Cx43 phosphorylation in major diseases of the cardiovascular system and the nervous system and its influence on Cx43 HCs and Cx43 GJIC. Cx43, Connexin 43; HC, hemichannel; GJIC, gap junction intercellular communication; ↑, Increase; ↓, Decrease.

Cx43 phosphorylation regulates the opening and closing of gap junction channels and hemichannels. During a steady state, cultured astrocytes exhibit that gap junctional communication is active at a high level, while hemichannel activity is modest [14]. In contrast, depolarization, ischemia, specific cation mobilization, and connexin phosphorylation activate the hemichannel, resulting in the astroglial sustained release of excitatory L-glutamate, D-serine, adenosine triphosphate, kyonurenine metabolites, and eicosanoids [2,15,16]. At several stages of the connexin ‘life cycle’, phosphorylation plays a role in gap junctional cell-to-cell communication (GJIC), including trafficking, assembly/disassembly, and the gating of gap junction channels [17,18]. The phosphorylation of Cx43 is a fundamental regulation process during disease progression. Many growth factors, oncogenes, and tumor-promoting chemicals can phosphorylate Cxs and may be involved in the occurrence and development of depression, cerebral ischemia, arrhythmia, neurodegenerative diseases, and other diseases [19] (Figure 1). Additionally, it has been shown in various systems that a number of important serine phosphorylation sites are crucial for mediating both slow and quick changes in Cx43 expression or plaque stability. For instance, the ubiquitin protein ligase Nedd4 binds the gap junction-specific protein Cx43 in living organisms. The interaction between Nedd4 and Cx43 does not require the Ser279/Ser282 phosphorylation of Cx43. Instead, the binding of both proteins may be modulated by the Cx43 phosphorylation status. The interaction of the two proteins definitely affects both the internalization of gap junctions and the post-internalization degradation of the gap-junction proteins [20]. Thus, the molecular mechanism of Cx43 phosphorylation is of great importance.

## 2. Functional Significance of Phosphorylation of Cx43 in the Nervous System

### 2.1. Changes in Cx43 Phosphorylation Associated with Depression

**Figure 2:**
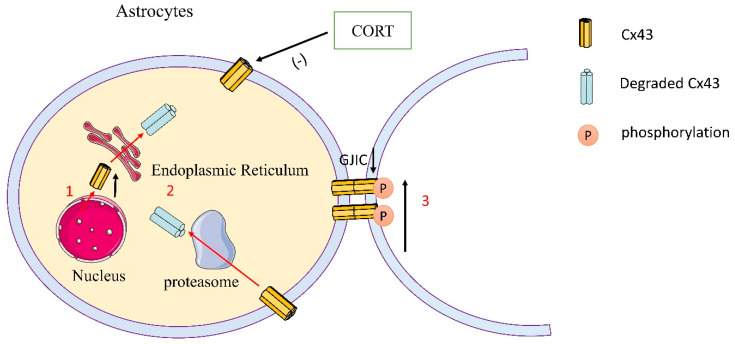
Schematic representation of Cx43 expression and phosphorylation of astrocytes in depression. (1) CORT reduced the synthesis of Cx43 and the distribution of Cx43 in the membrane in astrocytes. (2) Cx43 was degraded by a proteasome. (3) CORT reduced the distribution of Cx43 in the membrane and in the meantime increased the phosphorylation of Cx43 at Ser368 in the prefrontal cortical astrocytes and hippocampal astrocytes, thereby inhibiting the communication mediated by gap junctions.

Among many types of connexins, Cx43 is a major component of gap junctions in the brain, expressed primarily in astrocytes, and is required for astrocyte-mediated neuroprotection. Accumulating evidence suggests that astrocytes play an important role in the pathogenesis of major depression disorder (MDD). In particular, gap junction dysfunction in astrocytes is a potential target for MDD therapy. Several lines of evidence suggested the mechanism of stress-induced gap junction dysfunction in the prefrontal cortex and hippocampal astrocytes. Additionally, they experimentally demonstrated that corticosterone (CORT) disrupted the function of gap junctions due to the reduced distribution of Cx43 on the cell membrane and enhanced the phosphorylation of Cx43 at the Ser368 site [21]. When patients with depression undergo in vitro therapy, CORT simulates high levels of glucocorticoids. Previous data showed that Cx43 and Cx30 were significantly downregulated in patients with depression and in animal models of depression [22,23,24,25,26]. In addition, Cx43 mRNA has decreased in depressed patients [22,23,25,27]. In addition, several postmortem investigations showed that major depressive patients’ locus coeruleus, frontal cortex, mediodorsal thalamic nucleus, and caudate nucleus Cx43 expression was diminished in comparison to healthy people [22,23,24,25]. Moreover, it has been demonstrated that alterations in Cx43 expression in astrocytes are closely related to the development of various neuropathological diseases, including epilepsy, ischemia, Parkinson’s disease, and Huntington’s disease [28,29,30]. Furthermore, a recent study showed that interfering with cell communication by downregulating Cx43 gap junctions in the rat prefrontal cortex induces depression-like behaviors [26]. Studies have shown that Cx43 phosphorylation at Ser368 is elevated in both the corticosterone-exposed prefrontal cortex and hippocampal astrocytes. [31,32,33]. Another study found that the phosphorylation of Cx43 at Ser368 induced the internalization and degradation of gap junctions [34] (Figure 2). It has been demonstrated that the monomer ginsenoside Rg1 used in traditional Chinese medicine has antidepressant properties. By reducing the malfunctioning of gap junction channels (GJCs), Rg1 has antidepressant effects. It is possible that the improved function of GJCs may be due to Rg1 reversing the increased phosphorylation of Cx43 at Tyr265 and Ser279 sites induced by CORT [35]. Chronic unpredictable stress can increase C-Src expression. C-Src can directly phosphorylate Cx43 at Tyr247 and Tyr265 to cause the closure of the GJs channel [36]. Combined with these findings, targeting Cx43 phosphorylation to modulate gap junction function may be an effective approach for the treatment of MDD.

### 2.2. Changes in Cx43 Phosphorylation in Neurodegenerative Diseases

#### 2.2.1. Alzheimer’s Disease

**Figure 3:**
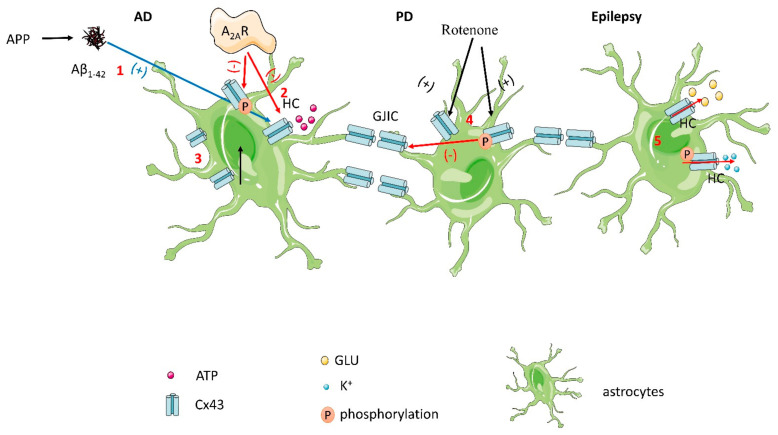
(1) In Aβ_1–42_-stimulated astrocytes, phosphorylation of Cx43 Ser368 residues is enhanced, resulting in a surge in hemichannel activity and ATP release. (2) A_2A_R directly regulates hemichannel activity and inhibits the upregulation and phosphorylation of Cx43 in Aβ_1–42_-exposed astrocytes. (3) Cx43 expression is increased in AD patients. (4) In rotenone-treated cultured astrocytes, Cx43 protein levels and phosphorylation levels were enhanced, and phosphorylated Cx43 led to gap junction cell-to-cell communication dysfunction. (5) In epilepsy, phosphorylation of Cx43 results in the release of K+ and glutamate, which increases connection conductance.

Cx43 has been reported to be involved in developing certain neurodegenerative diseases, including Alzheimer’s disease, Parkinson’s disease, and epilepsy [37,38] (Figure 3). The prominent pathological feature of Alzheimer’s disease (AD) is the formation of amyloid plaques, which are deposits of different sizes of small peptides called β-amyloid (Aβ). These Aβ plaques are derived via sequential proteolytic cleavages of the Aβ precursor protein (APP) by the action of β- and γ-secretases. Among the different Aβ fragments, Aβ_1–40_ and Aβ_1–42_ are thought to be critical elements in AD pathogenesis due to their neuropil and vascular accumulation. Researchers pointed out that AD brains showed the upregulation of A_2A_R and Cx43, which form hemichannels in astrocytes that can mediate ATP release [39]. A_2A_R directly regulates hemichannel activity and inhibits the upregulation and phosphorylation of Cx43 in Aβ_1–42_-exposed astrocytes. While hemichannels can open under resting conditions to facilitate basal paracrine communication, they are generally believed to become more active under pathological conditions [14,40,41,42,43,44]. The activity of hemichannels is highly regulated by connexin phosphorylation. In particular, Cx43 can be phosphorylated by several kinases, such as (PKC), protein kinase A (PKA), cyclin-dependent kinase p34cdc2, and casein kinase 1 [45,46,47]. Notably, Cx43 levels were upregulated in astrocyte processes near Aβ plaques in both animal AD models and brain samples from human AD patients [48,49,50,51]. A_2A_R regulates Cx43 levels and phosphorylation in Aβ_1–42_-exposed astrocytes. Connexin phosphorylation at Ser 368 can modulate hemichannel activity in astrocyte cultures [6,52]. Furthermore, extracellular ATP-derived adenosine plays an important role in the maintenance of A_2A_R overactivation in the Aβ_1–42_-induced upregulation of Cx43 levels and phosphorylation status. The extracellular conversion of ATP to adenosine by CD73 maintains the A_2A_R-mediated increase in Cx43 hemichannel activity to support the further release of ATP and other astrocyte signals that contribute to neurodegeneration. Astrocyte Cx43 hemichannels are increasingly recognized as relevant mediators of memory impairment in AD patients. Previous research is the first to demonstrate that the A_2A_R-mediated control mechanism of Cx43 activity in astrocytes may be related to the phosphorylation of Cx43, a well-established mechanism for regulating Cx43 hemichannel activity [44,46,53,54]. In Aβ_1–42_-stimulated astrocytes, the phosphorylation of Cx43 Ser368 residues was enhanced, and a surge in hemichannel activity and ATP release was observed. Studies have shown that the phosphorylation of connexin 43 increases at week 42 of AD, which is consistent with the dysregulation of gap junctions and the activation of astrocytes [55]. The study identified two sites of increased phosphorylation on Cx43, Ser365, and Ser368. Increased phosphorylation at Ser368 is functionally associated with a decrease in gap junction permeability and is generally associated with decreased traumatic brain injury (TBI) regions and glutamate clearance [56]. Therefore, targeting astrocyte Cx43 hemichannels may be a promising therapeutic approach for AD.

#### 2.2.2. Parkinson’s Disease

Recent evidence revealed that the major connexin in astrocytes, connexin 43, was enhanced in a rotenone-induced model of rat Parkinson’s disease (PD) [57]. In rotenone-treated cultured astrocytes, the enhancement of Cx43 protein levels paralleled the increase in gap junction cell-to-cell communication but was not accompanied by an increase in Cx43 mRNA levels. In addition, rotenone-induced increases in Cx43 protein levels were associated with increased levels of phosphorylated Cx43. In a rat model of PD, phosphorylated Cx43 was selectively enhanced in the basal ganglia region, which contains dopamine (DA) neurons or their terminal regions. Cx43 is primarily phosphorylated at Ser368 residues and exists in multiple isoforms after electrophoretic separation, non-phosphorylated (P0~41 kDa), or phosphorylated (P1~44 and P2~46 kDa) variants [58,59]. The phosphorylation levels of P0 and P1 were enhanced during the induction of Cx43 total protein by rotenone [58]. The findings suggest that the regulation of Cx43 protein phosphorylation in astrocytes, which leads to a reduction in gap junction cell communication, may play an important role in PD pathology.

#### 2.2.3. Epilepsy

The role of Cx43 GJs in epilepsy is complex and obscure. According to the literature, there are two possible theories: GJIC is thought to be critical for maintaining tissue homeostasis and propagating electrical and metabolic signals in cell populations [60]. Consistent with this, GJs can suppress seizure activity by redistributing K^+^ and glutamate (Glu) [61,62,63,64,65]. Astrocytes are electrically and metabolically connected to each other through gap junctions mainly composed of Cx43 and Cx30, forming a functional network [66,67]. It was found that coupling was completely absent in hippocampal tissue excised from patients with mesial temporal lobe epilepsy with hippocampal sclerosis (MTLE-HS). In contrast, coupled astrocytes were abundantly present in the hippocampus of non-sclerotic MTLE patients [68]. Recent reports using phospho-specific antibodies suggest that the phosphorylation of Ser365 is necessary for conversion to the P1 isoform [69]. In Co^2+^-induced epileptiform activity in hippocampal slices, a marked increase in P1 and a slight increase in P2 and P0 Cx43 have been reported [70]. The phosphorylation of Cx43 at Ser255 and/or 368 was enhanced. Phosphorylation at Ser255 and Ser368 of the Cx43 c-terminal reduces junctional conductance. The phosphorylation of MAPK at Ser255 reduces the open probability of Cx43 channels, whereas Ser368 is targeted by PKC, resulting in an increased open probability [4,71]. Taken together, the redistribution of the Cx43 protein and/or the alteration of its C-terminal phosphorylation may contribute to the uncoupling of MTLE-HS. Importantly, it provides new insights into the molecular mechanisms underlying astrocyte dysfunction in epilepsy, which may be key to the development and progression of epilepsy disorders.

### 2.3. Variations in Cx43 Phosphorylation in Conditions Affecting Cerebral Blood Flow

#### 2.3.1. Ischemic Stroke Induces the Occurrence of Cx43 Phosphorylation

The function of Connexin 43 is influenced by kinases that phosphorylate specific serine sites near its C-terminus. Stroke is a powerful inducer of kinase activity. Researchers studied wild-type (WT) and knock-in Cx43 at PKC site Cx43 Ser368, casein kinase 1 (CK1) site Cx43 Ser325/328/330, and MAPK site Cx43 Ser255/262/279/282 (MK4) in a permanent middle cerebral artery occlusion (pMCAO) stroke model [72]. The results of the research showed that astrocyte Cx43 gap junction channels (GJCs) affect neuronal survival under ischemic conditions [73,74,75]. The MAPK phosphorylation levels were more pronounced in WT and MK4 astrocytes when stressed in vitro for 3 h in ischemia buffer. Under normal circumstances, astrocyte Cx43 can exist in hemichannels or couple with other hemichannels on astrocytes, neurons, or oligodendrocytes to form glial syncytia, which are involved in the interaction of communication cells. The exchange of metabolites between them, thereby, maintains the nervous system environment. In ischemic stroke, the phosphorylation of Cx43 may lead to the degradation of gap junctions and the opening of hemichannels, thereby promoting the release of inflammatory mediators. The remaining gap junctions can facilitate the exchange of protective and harmful metabolites between healthy and damaged cells, protecting damaged cells to some extent. Various studies have shown that the phosphorylation state of the astrocyte Cx43 C-terminus is an important mediator of the regulation of gap junction channels and hemichannels after ischemic stroke, thereby affecting astrocyte and neuronal function [76,77]. It has been reported that the C-terminus of Cx43 in astrocytes after ischemic stroke can be phosphorylated by a variety of protein kinases, including protein kinase C, mitogen-activated protein kinase (MAPK), pp60Src kinase, and casein kinase 1δ, inducing Cx43 internalization and further promoting the uncoupling process of astrocytes [18,78,79,80,81,82]. Interestingly, other studies have found that hypoxia in vitro may lead to C-terminal dephosphorylation of astrocyte Cx43, accompanied by astrocyte uncoupling [52,82]. These studies suggest that the gap junction uncoupling process in astrocytes is intermediate between the phosphorylation and dephosphorylation of Cx43 and may be the result of Cx43 phosphorylation and the cause of hemichannel Cx43 dephosphorylation. Furthermore, hemichannel opening induced by Cx43 dephosphorylation in astrocytes promotes the release of inflammatory mediators and increases neuroinflammation after ischemic stroke [65,83]. In ischemic stroke, the phosphorylation of astrocyte Cx43 may lead to the uncoupling of gap junctions between astrocytes and other parenchymal cells, thereby reducing the direct communication between these cells. The subsequent dephosphorylation of Cx43 on hemichannels activates hemichannel opening, promoting the release of various proinflammatory mediators and toxic molecules, such as ATP and Glu.

#### 2.3.2. Phosphorylation of Cx43 in Cerebral Ischemia

The process of cerebral ischemia/reperfusion (I/R) injury is often accompanied by the enhancement of gap junction (GJ) function and the increase in gap junction protein expression. It has been reported that in the event of I/R injury, the increased expression of Cx43 increases the release of Glu, ultimately leading to the injury of astrocytes [84]. There is a growing body of literature that reveals that Cx43 expression is abnormally elevated when the brain is damaged by ischemia, and the inhibition of Cx43 expression can improve brain I/R injury [85]. During cerebral ischemia-reperfusion, normal cells provide energy support and signal transmission to neighboring cells through gap junctions to reduce cell damage. In the ischemic brain, the expression of Cx43 in activated astrocytes is drastically decreased, followed by rapid dephosphorylation and internalization of superficial protein epitopes, leading to reduced neuroprotective effects of gap junctions [86]. Studies have shown that Cx43 localization and phosphorylation are significantly regulated during ischemia and injury [87,88]. In addition, increased Cx43 and elevated levels of Cx43 phosphorylation have been detected after ischemia induction [89]. These results not only confirm the formation of Cx43 heteromeric channels but also suggest that Cx43 and phosphorylated-Cx43 heteromeric channels are involved in brain injury during ischemia. Previous research has established that astrocytes mainly contain phosphorylated Cx43 and that these undergo dephosphorylation after hypoxia [80]. In a recent study, researchers found that the phosphorylation of Cx43 was increased in the brain after ischemia. The membrane Cx40/Cx43 and Cx40/phospho-Cx43 complexes have been shown to be increased during ischemia [90]. Heteromeric channels are reported to be inconsistent with homo-polymeric channels. Heteromeric channels display strongly asymmetric voltage-dependent gating responses. Therefore, increased Cx40/Cx43 and Cx40/phospho-Cx43 heteromeric phosphor-Cx43 membrane may contribute to the changes in gap junctions after ischemia induction that can lead to brain damage. Furthermore, the degradation of Cx43 during cerebral ischemia is caused by selective autophagy, thereby affecting neuroinflammation and apoptosis. It has been found that Cx43 is phosphorylated at Ser368, Tyr247, and Tyr265 during cerebral ischemia [91]. The phosphorylation of Cx43 is a mechanistic initiator leading to its autophagic degradation during cerebral ischemia. In HS (vascular dementia due to cerebral ischemia, hypoperfusion) with hippocampal sclerosis, Cx43 is characterized by enhanced C-terminal phosphorylation affecting channel permeability and enhanced phosphorylation at Ser255 and/or 368. Phosphorylation at positions Ser255 and Ser368 of the Cx43 C-terminal reduces junctional conductance.

#### 2.3.3. Phosphorylation of Cx43 in Cerebral Vasospasm

Cerebrovascular disease is the second leading cause of death among Chinese citizens, among which cerebral vasospasm caused by spontaneous subarachnoid hemorrhage is the main factor regarding the high disability and mortality rates. Recent evidence suggested that the phosphorylation of Cx43 might play an important role in cerebral vasospasm after subarachnoid hemorrhage [92,93]. The authors found that in a rabbit model of secondary subarachnoid hemorrhage, the phosphorylation level of Cx43 in the cerebral basilar artery was significantly increased compared with the normal group, and it showed a chronological change, whereas the dephosphorylation was the opposite. At the same time, the basilar artery angiography in rabbits revealed that its contraction was positively correlated with the phosphorylation level of Cx43, which further indicated that the phosphorylation of Cx43 was related to cerebral vasospasm. In addition, PKC activity was noticeably increased in the membrane fraction of vasospastic cerebral arteries, and PKC controls GJIC spatially and dynamically by phosphorylating Cx43 at Ser368 [94].

### 2.4. Cx43 Phosphorylation and Its Associated GJs Play Important Roles in Cancer

All the available data suggest that Cx43 and its associated GJs play an important role in cancer. The expression level of Cx43 showed a downward trend and increased levels in malignant tumors, especially in astrocytomas. GJ intercellular communication activity in glioma cells can be regulated by Cx43 phosphorylation and by combining Cx43 and its related proteins [95]. Cx43 can enhance the motility and invasiveness of astrocytoma cells. It also affects the separation of glioma cells from the tumor core into the peritumoral neocortex [96]. Decreased GJIC in tumor cells is often associated with decreased Cx43 expression [97]. Cx43 can be the potential target of therapy for malignant gliomas. In addition, the epidermal growth factor receptor (EGF receptor) signal transduction pathways are intricately involved in various processes that contribute to the development of malignancies, such as cell cycle progression, the inhibition of apoptosis, angiogenesis, tumor cell motility, and metastasis [98]. EGF has been shown to induce Cx43 serine phosphorylation and the inhibition of gap junctional intercellular communication in cell lines derived from multiple tissue types [99,100].

## 3. Functional Significance of Cx43 Phosphorylation in the Cardiovascular System

Cx43 is the major ventricular gap junction protein, but it is also expressed in atrial and endothelial cells. Numerous studies have shown that post-translational phosphorylation of Cx43 severely affects intercellular coupling through gap junction remodeling under pathological conditions. While many kinases have been shown to phosphorylate Cx43, only Akt, MAPK, and PKC have been shown to modulate the activity of the Cx43 hemichannel, potentially affecting cardiac excitability. For example, the phosphorylation of AKT, also known as protein kinase B (PKB), on Ser373 increases hemichannel function by increasing the levels of surface Cx43, while PKC inhibits hemichannel activity through the phosphorylation of Ser368 [101,102,103,104,105,106,107]. Src is a tyrosine kinase known to both phosphorylate Cx43 (residues Tyr247 and Tyr265) and affect gap junction intercellular communication. In diseased cardiac tissue, in which Src is active, an increase in intercalated disc and intracellular localized Cx43 phos-Tyr313 has been observed [108].

### 3.1. Cx43 Phosphorylation and Dephosphorylation Are Associated with Cardiac Ischemia/Reperfusion Injury

Myocardial ischemia leads to the dephosphorylation of Cx43 and the redistribution of gap junction components away from intercalated discs. In this way, the Cx43 protein loses its supportive role in coordinating contractile activation and triggers arrhythmias in the ischemic heart [109]. Furthermore, the repositioned Cx43 can operate as an open hemichannel. By blocking these channels to prevent the loss of key metabolites, the store ion imbalance, hindered cell swelling, and myocardial ischemia/reperfusion injury can be prevented [110,111]. Prolonged ischemia/hypoxia (>15 min) has been reported to induce sarcolemmal redistribution of Cx43 in isolated rat and rabbit hearts [112,113]. The dephosphorylation of Ser365 occurred rapidly (5 min) after ischemia, followed by increased phosphorylation of Ser368. This phenomenon is consistent with the ‘gatekeeper’ concept that Ser365 phosphorylation prevents Ser368 phosphorylation, resulting in an inverse relationship in vivo [114,115]. Therefore, Cx43 phosphorylation is essential in regulating hypoxia-induced cardiac injury.

### 3.2. Cx43 Phosphorylation in Hypertension and Cardiac Hypertrophy

The conclusions of studies related to Cx43 phosphorylation in hypertension are controversial. For instance, a recent study found that Cx43 expression was upregulated and Cx43 phosphorylation levels were increased [116,117,118]. However, Cx43 expression decreased in some studies [119,120]. It has also been suggested that some of the observed differences may be related to the degree of left ventricular hypertrophy associated with hypertension because in the human heart, mild hypertrophy increases, while extensive hypertrophy decreases left ventricular Cx43 expression [121]. Increased Cx43 protein levels and enhanced Cx43 phosphorylation in early cardiac hypertrophy have been observed. The prevention or attenuation of maladaptive myocardial Cx43 remodeling and dysfunction caused by hypertension may reduce the risk of arrhythmic death and heart failure [84]. Furthermore, all the studies on diabetes and hypercholesterolemia showed increased Cx43 phosphorylation [122,123].

### 3.3. Cx43 Phosphorylation in Heart Failure and Arrhythmia

In some heart failure studies, Cx43 was dephosphorylated and the phosphorylation level of the Ser255 residue of Cx43 was increased [124,125]. Decreased phosphorylation of Cx43 at Ser365 (PKA-dependent site) or Ser325/328/330 (CK1δ-dependent site) results in slowed cardiac conduction and enhanced arrhythmia [126,127,128]. In addition, studies have shown that chronic heart disease and arrhythmias, including cardiac hypertrophy and heart failure, are associated with decreased total Cx43 but increased dephosphorylated Cx43 [129]. Restricted cell-to-cell communication, hampered action potential propagation, and the emergence of cardiac arrhythmia are all a result of Cx43 being phosphorylated or dephosphorylated by p38. Additionally, p38 inhibition enhances cell-to-cell communication and diminishes arrhythmia occurrence [130]. Studies in wild-type and transgenic mice show that enhanced CK1 phosphorylation by Cx43 protects against arrhythmias [83].

## 4. Functional Significance of Cx43 Phosphorylation in Other Tissues

### 4.1. Cx43 Phosphorylation in Endothelial Tissue

Connexins expressed in the vasculature include Cx37, 40, 43, and 45 [131]. Cx43 phosphorylation has also been shown to change during atherogenesis, in which minimally modified low-density lipoprotein accumulates in the vessel wall to form plaques [132]. In a mouse model of atherosclerosis, the total Cx43 expression was decreased in the carotid arteries, whereas the phosphorylation levels of Ser368 and Ser279/282 were significantly increased [133].

### 4.2. Cx43 Is Expressed in Many Epithelial Tissues

The phosphorylation of Cx43 at Ser368 has been detected in the intestinal epithelial cells of MDR1α-deficient mice [134]. In normal adult mammary glands, Cx43 phosphorylated at Ser279/282 was detected in myoepithelial cells [135]. Cx43 plays a role in the repair of damaged organs in the epidermis by being phosphorylated by various kinases. It was also observed that the phosphorylation of Cx43 at Ser368 was specifically elevated in the basal cell compartment proximal to the wound edge. The increase in phosphorylation levels reached a maximum level at 24 h post-injury and returned to baseline levels by 72 h [136].

### 4.3. Phosphorylation of Cx43 as a Role in Proliferative Retinopathy

Cx43 plays a critical role in astrocyte apoptosis and the resulting preretinal neovascularization in a mouse model of hypoxia-induced retinopathy. The increased coupling in response to hypoxia is due to the phosphorylation of Cx43 by CK1δ. The inhibition of this phosphorylation with a CK1δ inhibitor or in site-specific phosphorylation-deficient mice similarly protected astrocytes from hypoxic injury [137]. This study suggests that targeting Cx43 phosphorylation in astrocytes is a potential treatment for proliferative retinopathy. The inhibition of Cx43 Ser325/328/330 phosphorylation enhances retinal revascularization and improves pathological neovascularization.

### 4.4. Phosphorylation of Cx43 and Chronic Pain

Chronic pain, including inflammatory, neuropathic pain, is a serious clinical issue. Cx43 phosphorylation has been demonstrated in chronic pain models. After sciatic nerve chronic constriction injury (CCI), increased phosphorylation at Ser368 of spinal astrocytic Cx43 was observed during the maintenance phase (10–20 days after CCI), and this response was induced by the downregulation of K-ATP channels [138]. Furthermore, increased phosphorylation at Ser368 of spinal astrocytic Cx43 has been reported in the bone cancer pain model. Although it was also reported that phosphorylated Cx43 is involved in mechanical allodynia through the production of chemokine CXCL12, the mechanism mediating phosphorylated Cx43-induced CXCL12 production was not elaborated [139,140].

## 5. Conclusions and Perspectives

The most abundant connexin in the brain and heart is Cx43. Cx43 phosphorylation can affect changes in Cx43-interacting protein binding, subsequently affecting the underlying signaling pathways of gap junction communication, hemichannel function, kinase activity, and cell biological function. The phosphorylation status of Cx43 at various serine, threonine, and tyrosine sites can significantly affect the expression and function of Cx43 in various pathological conditions, such as depression, neurodegenerative disease, cerebral ischemia, and myocardial ischemia (Table 1). In the future, the phosphorylation of Cx43 can be used as an emerging target to study its regulation on the nervous system and the cardiovascular system and its significance for the occurrence and development of diseases.

## Figures and Tables

**Table 1:** Summary table of the expression and phosphorylation of Cx43 in multiple diseases.

	Disease Types	Expression	Phosphorylation	Site	Function	References
The nervous system	Depression	↓	↑	Ser368, Ser279 Tyr247, Tyr265	GJCs↓	[31,32,33,34,35,36]
	AD	↑	↑	Ser368, Ser365	GJCs↓ HC↑	[55,56]
	PD	↑	↑	Ser368	GJCs↓	[58,59]
	Epilepsy	↑	↑	Ser255, Ser368	GJCs↓ HC↑	[69]
	Ischemic stroke	No significant change	↑	MK4, Ser368 Ser325/328/330	GJCs↓ HC↑	[77]
	Cerebral ischemia	↑	↑	Ser368, Tyr247 Tyr265	GJCs↓	[86,90,91]
	Cerebral vasospasm	↑	↑	Ser368	GJCs↓	[94]
	Cancer	↓	↑	serine	GJCs↓	[97,99,100]
The cardiovascular system	Cardiac ischemia/reperfusion injury	——	↑	Ser368	GJCs↓	[114,115]
	Hypertension and cardiac hypertrophy	↑	↑	——	GJCs↓	[116,117,118,119,120,121,122,123]
	Heart failure and arrhythmia	↓	Ser255↑ Ser365, Ser325/328/330↓		GJCs↓	[124,125]
Other tissues	Endothelial tissue	↓	↑	Ser368 Ser279/282	——	[133]
	Epithelial tissues	——	↑	Ser368 Ser279/282		[135,136]
	Proliferative retinopathy	——	↑	Ser325/328/330	——	[137]
	Chronic pain	——	↑	Ser368	——	[138]

Cx43, Connexin 43; HC, hemichannel; GJIC, gap junction intercellular communication; ↑, Increase; ↓, Decrease.

## Data Availability

The data presented in this study are available upon request from the corresponding author.

## Abbreviations

Cx43	Connexin 43
CNS	Central nervous system
Ser	Serine
Tyr	Tyrosine
GJ	Gap junctions
GJIC	Gap junctional cell-to-cell communication
MDD	Major depression disorder
CORT	Corticosterone
AD	Alzheimer’s disease
Aβ	β-amyloid
APP	Aβ precursor protein
PKC	Protein kinase C
PKA	Protein kinase A
TBI	Traumatic brain injury
TPA	Tissue polypeptide antigen
PD	Parkinson’s disease
DA	Dopamine
MTLE-HS	Mesial temporal lobe epilepsy with hippocampal sclerosis
pMCAO	Permanent middle cerebral artery occlusion
CK1	Casein kinase 1
PKB	Protein kinase B
CXCL12	Chemokine (C-X-C motif) ligand 12
